# “Go Make Your Face Known”: Collaborative Working through the Lens of Personal Relationships

**DOI:** 10.5334/ijic.2574

**Published:** 2017-08-10

**Authors:** Nigel King, Alison Bravington, Joanna Brooks, Jane Melvin, David Wilde

**Affiliations:** 1 School of Human and Health Sciences, University of Huddersfield, Queensgate, Huddersfield HD1 3DH, UK; 2 Hull/York Medical School, University of Hull, UK; 3 Macmillan Cancer Support, 89 Albert Embankment, London SE1 7UQ, UK; 4 School of Social Sciences, Nottingham Trent University, Burton Street, Nottingham NG1 4BU, UK

**Keywords:** Collaboration, nursing, qualitative, inter-professional relationships

## Abstract

**Background:**

Collaborative working between professionals is a key component of integrated care. The academic literature on it largely focuses either on integration between health and social care or on the dynamics of power and identity between doctors and nurses. With the proliferation and extension of nursing roles, there is a need to examine collaborative working amongst different types of nurses.

**Method:**

This study explored experiences of collaborative working amongst generalist and specialist nurses, in community and acute settings. We carried out semi-structured interviews, incorporating the Pictor technique, with 45 nurses, plus 33 other key stakeholders. Transcripts were analysed using Template Analysis. This article focuses on one major thematic area that emerged from the analysis: the significance of interpersonal relationships amongst nurses, and between them and other professionals, patients and carers.

**Results:**

Relationship issues were ubiquitous in participants’ accounts of collaborative working. Good personal relationships facilitated collaboration; face-to-face interaction was especially valued. Relationships were recognized as requiring effort, especially in new roles. Organisational changes could disrupt productive personal networks.

**Conclusion:**

Relationship issues are integral to successful collaborative working. Policy and practice leaders must take this into account in future service developments. Further research into collaborative relationships in different settings is needed.

## Introduction

### Background

Collaboration between health and social care professionals has long been a concern of policy-makers worldwide [1], and is integral to integrated care [2,3,4]. Indeed, collaborative working features prominently in public policy beyond health and social care [5] and is increasingly the focus of attention in the private sector [6]. The ubiquity of collaborative working and the consequent need to understand what leads to effective and ineffective practice highlight the need to build the research base in this area. This is underlined by evidence that failures in collaborative working are an important factor in health and social care errors [7], including some very high profile cases, such as the case of the death following prolonged abuse of the British infant known as “Baby Peter” [8].

The research literature on collaborative working is broad and diverse, making it a challenge to obtain a clear overview of the field, which tends to be clustered around particular disciplinary, theoretical and/or topic interests. Additionally, there is a wide range of terms that overlap, but are not entirely synonymous with, collaborative working. These include multi-/inter-/trans-professional teamwork, interagency working, joint working, partnership working and so on. There is no consensus on the definitions of the individual terms.

Given this, it is important that we are clear about what we mean by “collaborative working” and how we see this as relating to other associated concepts. For the purposes of this article, we will use the following definition:

*Collaborative working occurs when two or more professionals from different professional groups are required to interact to ensure that appropriate care is delivered to a service user*.

There are two key aspects to this definition. Firstly, it portrays collaborative working as a broader concept than most other related terms. Unlike “teamwork” it is not restricted to what happens within teams, and may also refer to collaboration across teams, organisations and sectors. Similarly, it may occur as part of a formal initiative to achieve service integration, but it is not restricted to that context. It may involve collaboration between groups within as well as across professions. It embraces both long-term, highly integrated working practices and those that are more superficial and transient. Secondly, our definition has an explicit concern with interaction. Even when we are looking at collaboration on a macro-scale, a focus on collaborative working means a focus on how professionals relate to each other. This does not mean that we believe all research on collaborative working should be reduced to the exploration of interpersonal dynamics, but that whatever the level of analysis, it must be recognized that collaboration is always enacted in human interactions and relationships.

The importance of personal relationships in collaborative working is widely recognized in the literature [9], especially in relation to the need for trust, mutual understanding and shared goals and visions. Maxwell, Baillie, Rickard and McLaren [10] argue that successful establishment of new nursing roles depends on sharing social identities with co-workers, and highlight the role of interpersonal relationships in achieving this. D’Amour, Ferrada-Videla, San Martin-Rodriguez and Beaulieu [11] note the need for research that seeks to “…understand what transpires within the working lives of a group of collaborating professionals” (p.126). There is a substantial literature on inter-personal and inter-group dynamics within specific formally-constituted teams – especially surgical teams [12,13] – often concerned principally with issues of power and gender. However, as we have argued above, collaborative working has a much broader remit than this particular setting.

### Rationale for the present study

The topic of collaborative working has received less attention with regard to different nursing disciplines than it has in the context of working relationships between doctors and nurses or in health and social care integration. Our own previous research [14] suggests there can be significant challenges for such collaboration, especially given the proliferation of nursing roles in the last two decades in many parts of the world [15,16,17,18]. The integration of new and existing roles has not always been unproblematic. For example, the introduction of community matrons in the UK (a case-management role targeted at those with complex chronic health problems) faced significant local challenges in implementation [19] and in some specific areas of practice such as end-of-life care [14]. In Australia, a range of barriers to the development of advanced nursing roles has been identified [20] including lack of understanding of the role amongst other professionals. A meta-synthesis of studies examining the introduction of Nurse Practitioner roles across seven countries describes the journey as “tortuous” [21].

In the current qualitative interpretive study, we explored nurses’ experiences of collaborative working across sectors – acute and community settings, primary and secondary care, health and social services. We examined collaboration amongst different types of nurses (generalist and specialist), and also between nurses and other professionals, patients and carers. We also compared experiences in relation to providing care for cancer and for long-term condition (LTC) patients, though a detailed consideration of this comparison is beyond the scope of the present article.

## Theory And Method

### Theoretical position

The focus of this study was on collaborative working as it was experienced by nurses and other care professionals within their day-to-day lives. As such its approach was largely inductive and bottom-up, rather than seeking to impose a fixed theoretical framework upon data collection and analysis. However, it did draw upon key insights from our own and others’ previous work. These included constructivist notions of professional identity [22], which see people’s sense of what it means to be a particular kind of professional as rooted in interaction with others. This guided us towards a concern with interactions and relationships in particular cases, as captured by our use of the “Pictor” technique discussed further below [23]. We were also influenced by multi-level approaches to understanding collaboration, as expressed for example by D’Amour et al’s model [24]. Thus, although our starting point in data collection was with personal experience, this was always set in an understanding of the contexts of local services and wider national policy. Our inclusion of interviews with a range of stakeholders beyond our principle nursing groups – including senior managers from across sectors – helped us achieve this.

Philosophically, we would identify this research with what Hammersley [25] calls a “limited realist” position. This assumes a realist ontology, arguing that there is a common reality that exists outside our attempts to research it. However, it also accepts that it is not possible through research procedures to view this reality in a neutral and objective way; the researchers’ subjectivities will always shape the research process. Thus the epistemological position is relativist or constructivist. The aim in limited realist research is therefore not to definitively test hypotheses, but to build plausible and credible interpretations.

Given the lack of previous research on collaborative working amongst different groups of nurses, and our focus on how collaboration manifests within the everyday working lives of practitioners, our research question was framed in broad, exploratory terms:

How do different groups of nurses experience collaborative working in their everyday practice, with each other, other professionals, and patients and carers?

### Study setting

Our study location was a metropolitan borough in the north of England, centred on a large former industrial town. The borough is adjacent to several other highly populated urban areas. Life expectancy is lower than the national average, with significant variation between the most and least deprived areas. Morbidity and mortality rates are high for many common chronic illnesses. At the time of the study most of the borough was served by a single Acute Trust, with one main general hospital and several smaller facilities. Community nursing services were provided through a single Community Trust, though a minority of patients living close to boundaries accessed services in neighbouring areas. The Local Authority providing social care was effectively coterminous with the health Trusts. Data collection commenced in 2010, shortly after the new UK coalition government had announced plans for significant Health Service reforms, and continued into late 2012 when implementation of these was well underway.

### Sample and recruitment

The core participant groups were generalist and specialist nurses, working in acute or community settings. However, in order to obtain a rounded picture of services, we also recruited participants from a range of other professional and managerial groups, as well as representatives of patients and carers. We identified nursing and other groups from which to recruit on the basis of previous research and initial discussions with senior managers across sectors, and our funders (Macmillan Cancer Support). For the community generalist nurses we recruited from teams based across different locations within the borough. We accessed these groups initially via their line managers; once their permission was obtained we sent recruitment packs to individual nurses, seeking where possible to obtain variety in terms of tenure and location. For the specialist nurses, we sought to recruit from a range of different teams, and from cancer and long-term condition specialisms. Other professionals were approached directly or through line managers as appropriate. Patients and carers were recruited via local support groups.

Ethical approval was obtained from the School of Human and Health Sciences Research Ethics Panel, and from the South Yorkshire Research Ethics Committee (ref 09/H1310/76).

Details of participant groups are provided in Table 1.

**Table 1:** Details of participants.

Participant type	Number

District nurses	15
Community matrons	11
Hospital ward nurses	2
Community LTC specialist nurses	7
Acute LTC specialist nurses	4
Acute cancer specialist nurses	4
Acute palliative care specialist nurses	2
Community managers	11
Acute managers	4
Cross-sector posts	1
GPs	2
Social services staff	3
Patients	6 (3 cancer, 3 LTC)
Carers	6 (3 cancer, 3 LTC)
*Total*	*N = 78*

### Interview design and procedure

Data were collected using semi-structured interviews, incorporating the “Pictor” technique [23], a visual method used for exploring experiences of collaborative working. We developed Pictor specifically to examine how professionals experienced day-to-day involvement in collaborative working, as we found that using conventional semi-structured interviews often led to rather idealized versions instead of accounts grounded in actual practice [26]. Participant-generated visual methods such as Pictor can help people focus on their direct personal experience of a particular incident or episode, in a way that can feel less interrogative than a typical interview [27,28].

**Figure 1:**
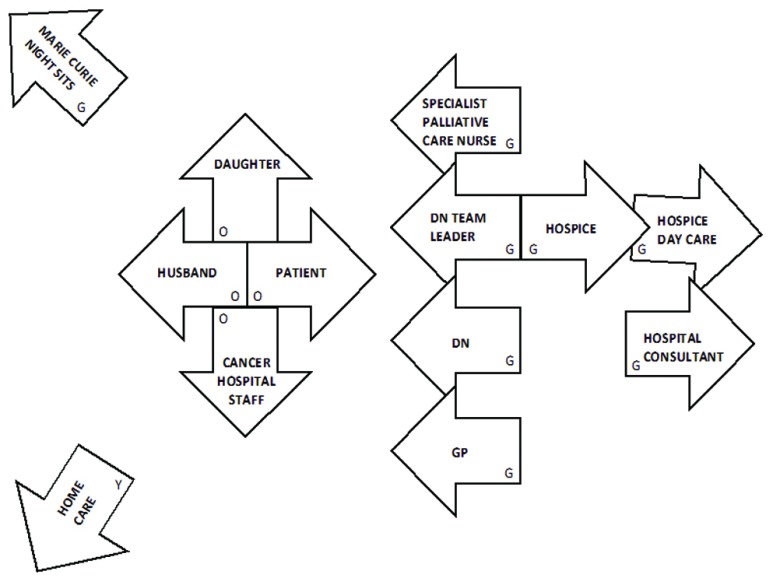
Example of Pictor chart, ‘Fiona’ (District Nurse), G = green arrow, O = orange arrow, Y = yellow arrow.

Figure 1 shows an example of a Pictor chart produced by one of our participants, ‘Fiona’ a district nurse (a generalist community nurse). The case she described is of a woman in her early forties with recurrent breast cancer and bone secondaries. Fiona has placed the arrows in two main groups. The patient is on the left with her husband, adult daughter and the staff of the cancer specialist hospital where she received treatment. These represent the people Fiona portrayed as the closest support for the patient. On the right is another group that represent what might be considered a ‘second line’ of support, which itself consists of two lines – the community services (including Fiona herself) nearer the patient group and hospice services behind them. Two arrows are placed to the left of the patient’s group at some distance – ‘Marie Curie night sits’ (a charity service) and ‘Home Care’. Both of these were only brought in very near the end of the patient’s life.

There are several features of the way Fiona uses the arrows that are interesting to note, in addition to the groupings described above. Firstly, she decided from the start to use the colour of the arrows to distinguish groups of agents on the chart, with professionals in green, family in orange and the single arrow for social care (‘Home care’) in yellow. Initially she also used a yellow arrow for the Marie Curie service, but asked the interviewer to change this to green:

It (Marie Curie) should have been in another colour but I just thought I’d put the family in one colour and the professionals in another and then social care…yeah, it should go in green, shouldn’t it?

Secondly, there was no suggestion that the direction of arrows represented anything about her experience and perception of particular agents, while many other participants did use it to indicate quality of relationships, conflict and so on. These points illustrate the importance of considering the use of the arrows in each case, rather than assuming what participants are indicating on the basis of what is “normally” done.

In a typical Pictor interview, the participant is asked to select a specific case that fits the criteria of the research study. They then think of all the people involved in the case and are invited to place the arrows on a large sheet of paper in a way that represents their perception of what happened in the case. For example, they may place some arrows close to the patient because they were the most frequently involved; they may place some pointing away from the patient to indicate an unsupportive attitude. However, there is no fixed way in which participants are required to use the arrows – they may do so in whatever way helps them to tell the story of the case. The “chart” created in this way is used as the basis for discussion of the participant’s experiences. We would normally anonymise it and then take a digital photograph of it as a record, to be referred to, either as a whole or in part, in analysis.

In the present study, each interview began by asking participants to provide a brief description of their role and professional experience. They were then asked to produce two Pictor charts – one focused on a cancer case and one on a non-cancer case. If they had relevant experience with only one of these disease types they would just produce a single chart. Patients and carers each independently produced a chart based on the patient’s experiences. The cases were each discussed in turn, exploring issues emerging in relation to collaborative working. In the concluding section of the interview, participants were asked about their hopes, fears and expectations for the future.

### Data analysis

Interviews were audio-recorded and transcribed verbatim. Transcripts were analysed using Template Analysis (TA) [29]. TA is a form of thematic analysis that involves the creation of an initial coding template based on close examination of a sub-set of data. This template is then applied to further data, and modified where necessary until all data relevant to the research question are coded to a final version. Template Analysis is recognized as an approach that works well in applied research, especially in relatively large qualitative studies where a range of different groups’ perspectives need to be examined [30]. It has been widely used both in health and in management/organizational research [31,32,33,34]. We carried out comparisons of blind coding by all members of the research team at several stages in this process, to ensure themes elicited were well-supported by the data. Throughout the analysis we referred back to Pictor charts to help us clarify participants’ perceptions of their experiences. We also looked at patterns of similarity and difference between groups in their Pictor charts.

The study as a whole was concerned with experiences of collaborative working in the context of providing care for patients with cancer and/or non-malignant long-term conditions. Our main focus was on the different nursing groups we included – generalist or specialist, acute-based on community-based. The perspectives of other professional groups, patients and carers mainly served to enhance our understanding of the context of collaboration within which nurses worked. In presenting our findings we therefore mostly focus on examples from nurses. As we have observed in previous studies [35,14], participants responded very positively to the task of constructing a Pictor chart, and we found the tool highly effective in facilitating their reflection on specific examples of collaborative working.

The majority of our coding related to themes directly addressing issues of collaboration, encompassed in an overarching theme of *What Affects Collaborative Working?* Other broad areas identified as contextually important to experiences of collaborative working were captured in three further overarching themes: *Condition-specific Involvement* (which addressed differences in the nature of practice between cancer and long-term conditions), *Survivorship* (i.e. how the growing number of patients living longer with cancer impacted on professionals’ work), and *NHS Reorganization* (both current and historical).

In this article our focus is mainly on one of the subthemes of *What Affects Collaborative Working* – namely, *Impact of Inter-personal Relationships*. We have chosen to address this in particular partly for the theoretical reasons outlined in our introduction but also because the issue of personal relationships permeated much of the discussion. The complexities of experiences of collaborative working meant that relationship issues often overlapped with other aspects, such as role definitions and inter-team dynamics, as well as the wider contextual themes, especially *NHS Reorganization*. These overlaps will be apparent in the examples below where we consider the findings in relation to interpersonal relationships.

## Findings

Findings related to our selected themes are detailed below. Pseudonyms are used for all direct quotes.

### Accessibility and availability

Participants emphasized how important it was that other professionals were readily accessible and available when needed. This was more than just a matter of getting hold of the right person at the right time for specific input to care. Rather, it was very often framed in terms of relational aspects of collaboration, most evidently in participants’ frequent comments on the value of face-to-face contact:

Over the phone, it depends on your communication skills, often things are forgotten. But face-to-face they’re brought to mind a little bit better, and if you’ve got a good relationship with somebody – another professional – then they know where you’re coming from in terms of patient referrals.

Sandra (district nurse)

Many participants stated how helpful it was to be located in close physical proximity to other professionals, or conversely attributed weaknesses in communication and collaboration to the lack of co-location. One of many examples of the benefits of co-location was given by a lymphoedema specialist nurse, Pauline. The nature of her role meant that she very often had to liaise with a wide range of other specialist and generalist nurses, many of whom shared a building with her:

Working here in this building has been a real bonus because I’m working alongside, you know, physically working next to other specialists: dermatologists and heart failure nurses, COPD.

Pauline (lymphoedema specialist nurse)

Proximity is important in cases like this because of the opportunities it offers for informal contacts, where information can be shared and relationships can be built. However, formal meetings can also be important as spaces in which inter-professional relationships can be fostered. An example from a hospital-based specialist palliative care nurse illustrates this:

Because we’re palliative care, we will work with all the tumours, all the site-specific nurses, specialist nurses, because as their patients come to be palliative, to end-stage, they’ll refer them on to us. So we go to everyone’s MDTs *[multi-disciplinary teams]* as well because we’ll pick patients up from there.

Veronica (palliative care specialist nurse)

Despite the widespread preference for face-to-face communication, many participants reported positively on the use of telephone and (less commonly) e-mail contact. This did rely on other professionals being readily available on the phone or electronically, and the participants having confidence that such contact would be welcomed. There was some tendency for hospital-based nurses to be more favourable towards remote communications than community-based nurses, which may reflect the fact that they were often contacting colleagues with whom they already had a good face-to-face working relationship.

### Shared history

This theme encompasses two overlapping sub-themes: *longevity of personal relationships* and *common professional background*. Participants described many instances where a shared history – in terms of a long-standing personal relationship, and/or a common professional background - facilitated good inter-professional relationships. Both types of shared history could enhance working relationships through mutual understanding. Shared personal history also enabled the building of trust, with potential benefits for smooth and efficient collaboration across professional and/or organisational boundaries. One community-based specialist nurse (diabetes) emphasized her close connections with the equivalent acute-base service, and how this facilitated collaborative working:

Because I’ve known [name] who’s the manager for so long we have a very close relationship, so that if I ever have any problems that I can’t manage I can refer to the Acute Trust for specialist care with the Consultants […] We have close links with all the services really, and I think because a lot of us have worked together over the years we know names and faces.

Alice (community diabetes specialist nurse)

The phrase “names and faces” is telling here, as it underlines the personal way in which this participant construes these relationships. Conversely, lack of a personal relationship could impede collaboration, as in the following instance from a community matron, describing conflict with a palliative care specialist nurse (PCSN):

I do know the other nurses [PCSNs], but this nurse I didn’t know. Sometimes it’s easier fighting your corner with someone that you do know, and who knows you, but I think with this one because we didn’t know each other, when we was both being assertive it came across as a bit of an argument.

Rosie (community matron)

*Common professional background* was most often referred to in conjunction with *longevity of personal relationships*. For example Gill, a community diabetes specialist nurse talks about how her background as a practice nurse (i.e. a nurse working in a GP practice) helps her in her interactions with current practice nurses:

I was a practice nurse for so long and we used to have regular studies and things, I know how they operate and I know most of the practice nurses […] so I know them personally, it’s a quick phone call to get something like this resolved.

Gill, (community diabetes specialist nurse)

The combination of understanding of role from personal experience and a personal shared history is cited quite often as facilitating good relationships. It is less clear whether the common role history in itself is seen as having such an effect, though Amy, a district nurse locality manager, suggests that absence of it can impede mutual understanding. She compares those community matrons who had previously worked in district nursing to those from other backgrounds, in terms of their ability to liaise well with district nurses:

We lost twelve of our [district] nurses to community matrons [i.e. they changed role] and because they knew district nursing I think they understood our role more; now, the ones who’ve come from different backgrounds didn’t always understand it.

Amy (district nurse locality manager)

Other participants make a similar point about the impact of community matrons’ prior role history.

### Making an effort

Many participants highlighted the effort they put in to building relationships with colleagues from other disciplines and/or other organisations. This was more than just responding positively to others; it involved pro-active strategies to develop positive interpersonal relations, underpinned by trust and respect. There was a strong sense that developing good relationships required nurses to “make an effort”, beyond the interactional requirements of their day-to-day roles. This is nicely illustrated by one participant’s ‘recipe’ for establishing new relationships in order to earn trust and respect:

Going and seeing ‘em. Lurk outside a doctor’s room. I’m always lurking down here. Go in and see the doctor. Nip over and see the district nurses. Go to the Hospice – I know the girls at the Hospice now. Go to Intermediate Care. You can’t go all the time, I don’t mean that, but go make your face known.

Emily (community matron)

As the community matron was a relatively new role, it is not surprising that several participants from this group spoke at some length about their efforts to build relationships. Nafisa, for example, discusses this in relation to the tensions that can exist between hospital and community-based services:

I think it depends on the individual [community] matrons and how prepared they are to sell the service and to talk to these people and build up a good relationship, and that’s what I’ve always tried to do, and it works very well then.

Nafisa, (community matron)

Participants also acknowledged and valued the efforts of others to get to know them:

One GP regularly e-mails me to talk about, to arrange meetings for one particular lady that we’ve got on the caseload, who’s proving difficult to manage, and he wants to meet on Monday to discuss – which is brilliant. Face-to-face is much better than going through Reception.

Sasha (district nurse)

It is interesting to note in many cases, including the examples from Emily and Sashsa (above), participants stress face-to-face contact as a crucial aspect of effective relationship-building, underlining the points made in relation to our first theme (accessibility and availability).

### (Not) “Stepping on toes”

Building and maintaining effective collaborative relationships was commonly portrayed as requiring a level of diplomatic skill – showing awareness of others’ personal and professional positions, and sometimes being willing to compromise. New services were particularly at risk of being seen to be “stepping on toes”; this was experienced by many of the community matrons, for example. A cancer specialist nurse, Susan, detailed how a new service came into conflict with its long-established equivalent in a neighbouring hospital, requiring considerable effort to turn the relationship in a more constructive direction:

Susan: It got to a head a bit, really, and we got together and had a bit of a, you know, sort of a – I don’t know what you’d call it?

Interviewer: Heart to heart?

Susan: Yeah, mediation – great word, great word – where we said listen, you know, certain things we weren’t quite happy about and so that was the start of a bit more harmony.

Failure of others to show consideration could lead participants to experience conflict over roles and in the worst cases an enduring breakdown in relationships between groups. One district nurse describes a dispute with another service which had attempted to “take over”, with no prior discussion, a patient that the team had been managing. The failure to negotiate roles so as to avoid “stepping on toes” led to lasting damage to relationships between the two services.

### Organisational change and its impact on relationships

The British NHS has undergone a series of major organisational changes over many years, in addition to numerous more narrowly focused changes in particular services. Our research took place just as the current UK Government announced radical plans for reforms to the NHS and this inevitably figured strongly in the interviews. The start of our data collection also coincided with the announcement of local changes which certainly provoked anxieties for many of our participants. The context of change and uncertainty was recognized by some participants as having direct implications for collaborative working. One way in which this was manifest was in power struggles at a senior level as different organisations (and their sub-systems) sought to secure their position in the anticipated ‘new world’. Collaboration on the ground was seen as happening in spite of, rather than in response to, the actions of senior management:

On the ground there’s such a willingness to work together, and people will get by despite some of the senior managers and not because of them, and you know at a higher level people are getting embroiled in ownership, power and finance and things like that, but on the ground people are generally working together with a genuine commitment.

Anna (manager)

The guarded optimism of this manager, that clinical staff were able to work around the disruption and conflict at a higher level, needs to be set alongside a widespread feeling of powerlessness, pessimism and even futility in the face of continual NHS changes. There were fears that changes could fragment certain services and have a negative effect on communications between agencies. These were mirrored by similar concerns from social services who had themselves experienced significant “re-structuring”. One participant from social services described how change in both sectors can disrupt established relationships and communication channels across sectors:

It’s hard for the nurses to keep up with it, with us restructuring, and who does what, and who’s responsible for this and who deals with this group of people […] and they’ve [health services] restructured as well, and they’ve -like some of them have merged, and some of them have been reduced. And the difficulty with that is you get hold of a name that you know you want to speak to, someone in a particular area, and *[previously]* you’d always ring THAT person for some advice.

Elsa (social services manager)

It should be noted that for those staff interviewed soon after the announcement of the proposed NHS changes – principally community nursing staff – uncertainty about details is very likely to have added to negative reactions to change. However, pessimism was by no means confined to this group or to the earlier interviews in the study.

## Discussion

Overall, our findings confirm the importance of personal relationships in collaborative working, and highlight some of the key aspects of relationships that were commonly perceived to be crucial for success. We concentrate here on two issues that emerged strongly from the analysis and that we feel have implications for future policy and practice developments: the value of face-to-face contact, and the notion of relationship formation and maintenance as requiring effort. We will consider both of these in the context of organizational change.

### Face-to-face contact

As shown above, participants frequently stated how valuable they found face-to-face contact with colleagues when working collaboratively. We did not routinely probe for this issue; it very much emerged from participants’ own concerns. As the use of technology to support remote electronic communication in health and social care increases [36] we need to consider why face-to-face interaction remains so important to professionals. We did not see any evidence that this phenomenon was due to any particular local organisational or geographic issues, though arguably the mainly urban and suburban character of the borough made face-to-face contact more of a realistic possibility than it would be in a remote rural area. However, the context of ongoing and impending changes to the NHS at all levels may well have heightened fears about the maintenance of face-to-face contact in valued relationships.

We would argue that the desire for face-to-face contact reflects something fundamental about the nature of collaborative working: that it is essentially a relational process. Research in many different organisational settings shows that interaction between professionals is not just a matter of exchanging the information needed to get the job done. Rather, it is often also about developing and maintaining relationships that will enable the parties to work together well over the longer term. For example, across a range of organisations, Nardi and Whittaker [37] found face-to-face communication to play a major role in developing social bonds that were important to organisational success. Physical presence enabled modes of interaction that were difficult or impossible to achieve in mediated communication, and also served as a symbolic expression of the value of the relationship with colleagues or clients. In the very different setting of health and social care, our findings suggest that face-to-face interaction plays a similar role. They parallel those of Conn et al [38] who examined interprofessional communication in General Internal Medicine across five hospitals in Canada, and highlighted the importance of the availability of senior physicians and the value of good, informal relationships for effective communication.

In the context of health and social care, the rapid growth in the use of information technology in communication has attracted a considerable amount of research. A large proportion of this, though, is focused on communication between health/social care professionals and service users rather than amongst professionals. For example, a recent Cochrane Review of the use of e-mail in communication between health professionals found only one trial that met their inclusion criteria [39]. In contrast, a similar review of trials examining the use of e-mail in patient/caregiver and health professional communication (published three years earlier) found nine studies [40]. Amongst surveys and qualitative studies there is also a predominance of those concerned with patient/caregiver – professional communication. Those that do examine inter-professional communication highlight a range of perceived benefits, including convenience [41,42], the chance to disseminate information across a wide network [43], and the ability to keep an audit trail of communication [44,45]. However, they also point to limitations and areas of concern. Not surprisingly, these encompass such things as privacy and confidentiality, training and the reliability of technology [46,47]. However, they also include relationship issues. For example, Gross et al [48] describe a case study where the introduction of an electronic health record system led to damage to mutual trust amongst members of the inter-professional team. A study of the use of e-mail between primary and secondary care in the UK found that while some practitioners said they had been able to build good relationships beginning with e-mail contact, others felt that they required a personal, face-to-face relationship before they could use e-mail effectively [42].

### Relationships as effortful

Our findings show that good collaborative relationships do not just happen; people need to devote effort over time to negotiate potential conflicts and establish trust. Power dynamics play a part here. Many of the stories told by participants about their willingness to “make an effort” were either where they were in new roles whose credibility had to be proved to others (e.g. community matrons) or were in the context of relationships with those in a more powerful position (e.g. nurses with doctors, or practitioners with managers). This is in keeping with a substantial body of literature that recognises the significance of power issues in any consideration of collaborative working [13,49].

We would certainly agree that collaborative working is infused with issues of power, but our findings suggest that willingness to put effort into relationships is not only a product of power relations. There were many instances described which were not associated with obvious power imbalances. Above all, participants’ willingness to make an effort highlights the value they placed on good personal working relationships. The wish to make working life more pleasant doubtless played a part in this, as did the need to establish professional status in some cases. But the valuing of relationships was also associated with a commitment to doing one’s job well for patients. Participants frequently described how good relationships enabled effective care to be provided through appropriate referrals, timely access to advice for patients and ‘signposting’ through the complexities of multi-agency care. The anxieties expressed in relation to ongoing changes to the NHS – and to the sense of continuous turbulence over the long-term – were clearly fueled in part by concerns about their impact on working relationships. Existing relationships could become disrupted and the effort involved in building them would need to be repeated in response to the requirements of new structures.

### Experiences of using Pictor

We found that the great majority of our participants engaged very well with the Pictor task. Indeed, as we have found in other studies, many commented on how insightful and enjoyable they found the activity. Our subjective experience as interviewers was that participants found the method helpful in focusing on specific examples of collaborative working, and that it enabled them to reflect on cases from a wide perspective; that is to say, they did not just concentrate on a few “main players” in the case. This is evidenced by the large number of individuals and agencies included on many charts.

## Conclusion

### Challenges

A qualitative study such as this does not seek generalizability, but rather strives to provide a depth of understanding that can inform theoretical development and provide insights transferable to other cases. The fact that we chose to carry out our research in a single geographical and administrative area may limit the extent to which it can contribute to these goals. Other settings may bring different factors to bear that were not prominent in our location – for example, issues of distance and travel time in remote rural areas, or particular large-scale concentrations of ethnic minority populations. However, advantages from concentrating on a single setting include a real depth of understanding of the local context, and common issues facing the professional groups we interviewed.

Similarly, the choice to place the different groups of nurses at the centre of our attention will have brought to the fore certain issues that were especially important to them, but may not have figured as strongly for other groups. For instance, doctors may not have felt such motivation to go out and proactively develop relationships with nurses, given what we know about power and status imbalances between these groups [50,51]. However, we feel that our main focus on generalist and specialist nurses was justified with regard to the significance of these staff in health care provision and the relative neglect of their perspective in the literature.

### Implications for policy and practice

Introducing and developing integrated care in health and social services commonly entails changes in organisational structures, relocations, and/or the introduction of new roles and new service providers [52,53]. All these have the potential to disrupt effective networks of personal relationships between staff who need to work together to provide good care to patients. Currie, Finn and Martin [15] note that policy-makers frequently fail to take into account the social context into which new roles are introduced. In the UK, recent and current changes to the NHS have undoubtedly been perceived as disruptive, both in community and acute settings [54,55]. It was notable in many of our interviews that beyond responses to specific changes there was a widespread sense of weariness with constant re-organisation and change over many years.

We understand that at a time when health services are facing major challenges, including financial constraints, relationships amongst professionals do not seem a priority. We would argue, though, that in such circumstances it is especially important to attend to inter-professional relationships, in order to enhance effective collaborative working. Making time and space for relationship-building and maintenance is not a luxury, but an essential. Research – including our own – suggests that there are strategies that organisations can adopt to achieve this. Joint training initiatives, task-focused inter-professional meetings (formal and informal), co-location and shared spaces for interaction may all help to nurture good relationships.

### Directions for future research

Further in-depth research into collaborative working relationships in different settings is needed, varying in terms of geographical and population characteristics and service provision. Evidence from such studies will enable scholars to develop theoretical models of collaborative working that better integrate a relational perspective. We are not arguing that relationships should be positioned to dominate all other facets of the phenomenon, but that researchers working at all levels need to consider collaborative working through the lens of inter-professional relationships. However large-scale a collaborative initiative might be, on the ground it always involves individual professionals working together (or failing so to do).

In the longer term research is needed that looks at the effect on patient outcomes of the quality and nature of inter-professional relationships. This is difficult to achieve not only in terms of identifying and measuring appropriate factors, but also because it is likely that any impact on patients may take a relatively long time to become discernable. Nevertheless, we need to find ways to address the question, and detailed qualitative work looking at patient perceptions and experiences of how professionals work together [56] can help inform future larger-scale work focused on patient outcomes.

## References

[1] Zwarenstein M, Goldman J, Reeves S (2009). Interprofessional collaboration: effects of practice-based interventions on professional practice and healthcare outcomes. Cochrane Database Systematic Review.

[2] Boon HS, Mior SA, Barnsley J, Ashbury FD, Haig R (2009). The difference between integration and collaboration in patient care: results from key informant interviews working in multiprofessional health care teams. Journal of Manipulative and Physiological Therapeutics.

[3] Kodner DL, Spreeuwenberg C (2002). Integrated care: meaning, logic, applications, and implications – a discussion paper. International Journal of Integrated Care.

[4] Chung VCH, Ma PHX, Hong LC, Griffiths SM (2012). Organizational determinants of interprofessional collaboration in integrative health care: systematic review of qualitative studies. PLOS One.

[5] Dickinson H, Sullivan H (2014). Towards a general theory of collaborative performance: the importance of efficacy and agency. Public Administration.

[6] Xue X, Shen Q, Ren Z (2010). Critical review of collaborative working in construction projects: business environment and human behaviors. Journal of Management in Engineering.

[7] Zwarenstein M, Reeves S (2006). Knowledge translation and interprofessional collaboration: where the rubber of evidence-based care hits the road of teamwork. The Journal of Continuing Education in the Health Professions.

[8] Haringey Local Safeguarding Children Board (2009). Serious case review: Baby Peter. Executive Summary.

[9] Pullon S (2008). Competence, respect and trust: key features of successful interprofessional nurse-doctor relationships. Journal of Interprofessional Care.

[10] Maxwell E, Baillie L, Rickard W, McLaren S (2013). Exploring the relationship between social identity and workplace jurisdiction for new nursing roles: a case study approach. International Journal of Nursing Studies.

[11] D’Amour D, Ferrada-Videla M, San Martin Rodriguez L, Beaulieu M-D (2005). The conceptual basis for interprofessional collaboration: core concepts and theoretical frameworks. Journal of Interprofessional Care.

[12] Collin K, Paloniemi S, Mecklin JP (2010). Promoting inter-professional teamwork and learning–the case of a surgical operating theatre. Journal of Education and Work.

[13] Lingard L, Espin S, Evans C, Hawryluck L (2004). The rules of the game: interprofessional collaboration on the intensive care unit team. Critical Care.

[14] King N, Melvin J, Ashby J, Firthm J (2010). Community palliative care: role perception. British Journal of Community Nursing.

[15] Currie G, Finn R, Martin G (2010). Role transition and the interaction of relational and social identity: new nursing roles in the English NHS. Organization Studies.

[16] Booth J, Hutchison C, Beech C, Robertson K (2006). New nursing roles: the experience of Scotland's consultant nurse/midwives. Journal of Nursing Management.

[17] Kaasalainen S, Martin-Misener R, Kilpatrick K, Harbman P, Bryant-Lukosius D, Donald F, Carter N, DiCenso A (2010). A historical overview of the development of advanced practice nursing roles in Canada. Nursing Leadership.

[18] Strivastava N, Tucker J, Draper E, Milner M (2008). A literature review of principles, policies and practices in extended nursing roles relating to UK intensive care settings. Journal of Clinical Nursing.

[19] Drennan V, Goodman C, Manthorpe J, Davies S, Scott C, Gage H, Iliffe S (2011). Establishing new nursing roles: a case study of the English community matron initiative. Journal of Clinical Nursing.

[20] McKenna L, Halcomb E, Lane R, Zwar N, Russell G (2015). An investigation of barriers and enablers to advanced nursing roles in Australian general practice. Collegian.

[21] Andregård A-C, Langland E (2015). The tortuous journey of introducing the Nurse Practitioner as a new member of the healthcare team: a meta-synthesis. Scandinavian Journal of Caring Sciences.

[22] King N, Ross A (2004). Professional identities and interprofessional relations: evaluation of collaborative community schemes. Social Work in Health Care.

[23] King N, Bravington A, Brooks J, Hardy B, Melvin J, Wilde D (2013). The Pictor Technique: a method for exploring the experience of collaborative working. Qualitative Health Research.

[24] D’Amour D, Goulet L, Labadie J-F, San Martin-Rodriguez L, Pineault R (2008). A model and typology of collaboration between professionals in healthcare organizations. BMC Health Services Research.

[25] Hammersley M, Hammersley M (1992). Ethnography and realism. What’s Wrong with Ethnography? Methodological Explorations.

[26] Ross A, King N, Firth J (2005). Interprofessional relationships and collaborative working: encouraging reflective practice. Online Journal of Issues in Nursing.

[27] Sheridan J, Chamberlain K, Dupuis A (2011). Timelining: visualizing experience. Qualitative Research.

[28] Wilson S, Cunnigham-Barley S, Bancroft A, Backett-Milburn K, Masters H (2007). Young people, biographical narratives and the life grid: young people’s accounts of parental substance use. Qualitative Research.

[29] Brooks J, McCluskey S, Turley E, King N (2015). The utility of template analysis in qualitative psychology research. Qualitative Research in Psychology.

[30] King N, Symon G, Cassell C (2012). Doing Template Analysis. The practice of qualitative organizational research: core methods and current challenges.

[31] Horrell B, Stephens C, Breheny M (2015). Capability to care: supporting the health of informal caregivers for older people. Health Psychology.

[32] Thomas CEL, Phipps DL, Ashcroft DM (2016). When procedures meet practice in community pharmacies: qualitative insights from pharmacists and pharmacy support staff. BMJ Open.

[33] Wang XL, Bowie D (2009). Revenue management: the impact on business-to-business relationships. Journal of Services Marketing.

[34] Wyatt M, Silvester J (2015). Reflections on the labyrinth: investigating black and minority ethnic leaders’ career experiences. Human Relations.

[35] Hardy B, King N, Firth J (2012). Applying the Pictor technique to research interviews with people affected by advanced disease. Nurse Researcher.

[36] Free C, Phillips G, Watson L, Galli L, Felix L, Edwards P, Patel V, Haines A (2013). The effectiveness of mobile-health technologies to improve health care service delivery processes: a systematic review and meta-analysis. PLoS medicine.

[37] Nardi B, Whittaker S, Hinds P, Kiesler S (2002). The place of face-to-face communication in distributed work. Distributed Work.

[38] Conn L, Reeves S, Dainty K, Kenaszchuk C, Zwarenstein M (2012). Interprofessional communication with hospitalist and consultant physicians in general internal medicine: a qualitative study. BMC Health Services Research.

[39] Goyder C, Atherton H, Car M, Heneghan C, Car J (2015). Email for clinical communication between healthcare professionals (Review). Cochrane Database of Systematic Reviews.

[40] Atherton H, Sawmynaden P, Sheikh A, Majeed A, Car J (2012). Email for clinical communication between patients/caregivers and healthcare professionals (Review). Cochrane Database of Systematic Reviews.

[41] Leong S, Gingrich D, Lewis P, Mauger DT, George J (2005). Enhancing doctor-patient communication using email: a pilot study. Journal of the American Board of Family Medicine.

[42] Sampson R, Barbour R, Wilson P (2016). Email communication at the medical primary–secondary care interface: a qualitative exploration. British Journal of General Practice.

[43] Thede L (2007). Networking via e-mail. Computers, Information and Nursing.

[44] Car J, Sheikh A (2004). Email consultations in health care: 1. scope and effectiveness. British Medical Journal.

[45] Car J, Sheikh A (2004). Email consultations in health care: 2. acceptability and safe application. British Medical Journal.

[46] Katzen C, Solan MJ, Dicker AP (2005). E-mail and oncology: a survey of radiation oncology patients and their attitudes to a new generation of health communication. Prostate Cancer Prostatic Disease.

[47] Virji A, Yarnall KS, Krause KM, Pollak KI, Scannell MA, Gradison M (2006). Use of email in a family practice setting: opportunities and challenges in patient- and physician- initiated communication. BMC Medicine.

[48] Gross A, Leib R, Tonachel A, Tonachel R, Bowers D, Burnard R, Rhinehart C, Valentim R, Bunnell C (2016). Teamwork and electronic health record implementation: a case study of preserving effective communication and mutual trust in a changing environment. Journal of Oncology Practice.

[49] Fawcett J (2014). Thoughts about collaboration – or is it capitulation?. Nursing Science Quarterly.

[50] Kreindler SA, Dowd DA, Star ND, Gottschalk T (2012). Silos and social identity: the Social Identity approach as a framework for understanding and overcoming divisions in health care. The Milbank Quarterly.

[51] Gallagher A, Bousso RS, McCarthy J, Kohlen H, Andrews T, Paganini MC, Abu-El-Noor NI, Cox A, Haas M, Arber A, Abu-El-Noor MK, Baliza MF, Padilha KG (2015). Negotiated reorienting: a grounded theory of nurses’ end-of-life decision-making in the intensive care unit. International Journal of Nursing Studies.

[52] Bardsley M, Steventon A, Smith J, Dixon J (2013). Evaluating integrated and community-based care: how do we know what works?.

[53] Evans JM, Baker GR, Berta W, Barnsley J (2013). The evolution of integrated healthcare strategies. Advances in Healthcare Management.

[54] Doherty C (2009). A qualitative study of health service reform on nurses’ working lives: learning from the UK National Health Service. International Journal of Nursing Studies.

[55] Haycock-Stuart E, Kean S (2013). Shifting the balance of care? A qualitative study of policy implementation in community nursing. Nursing Management.

[56] Hardy B, King N, Rodriguez A (2014). The experiences of patients and carers in the daily management of care at the end of life: findings from a phenomenological study. International Journal of Palliative Nursing.

